# Supplementary figures and images for: Electrochemical Enrichment and Isolation of Electrogenic Bacteria from 0.22 µm Filtrate
Source: Microorganisms. 2022 Oct 18;10(10):2051. doi: 10.3390/microorganisms10102051 (PMC9611719; doi:10.3390/microorganisms10102051)

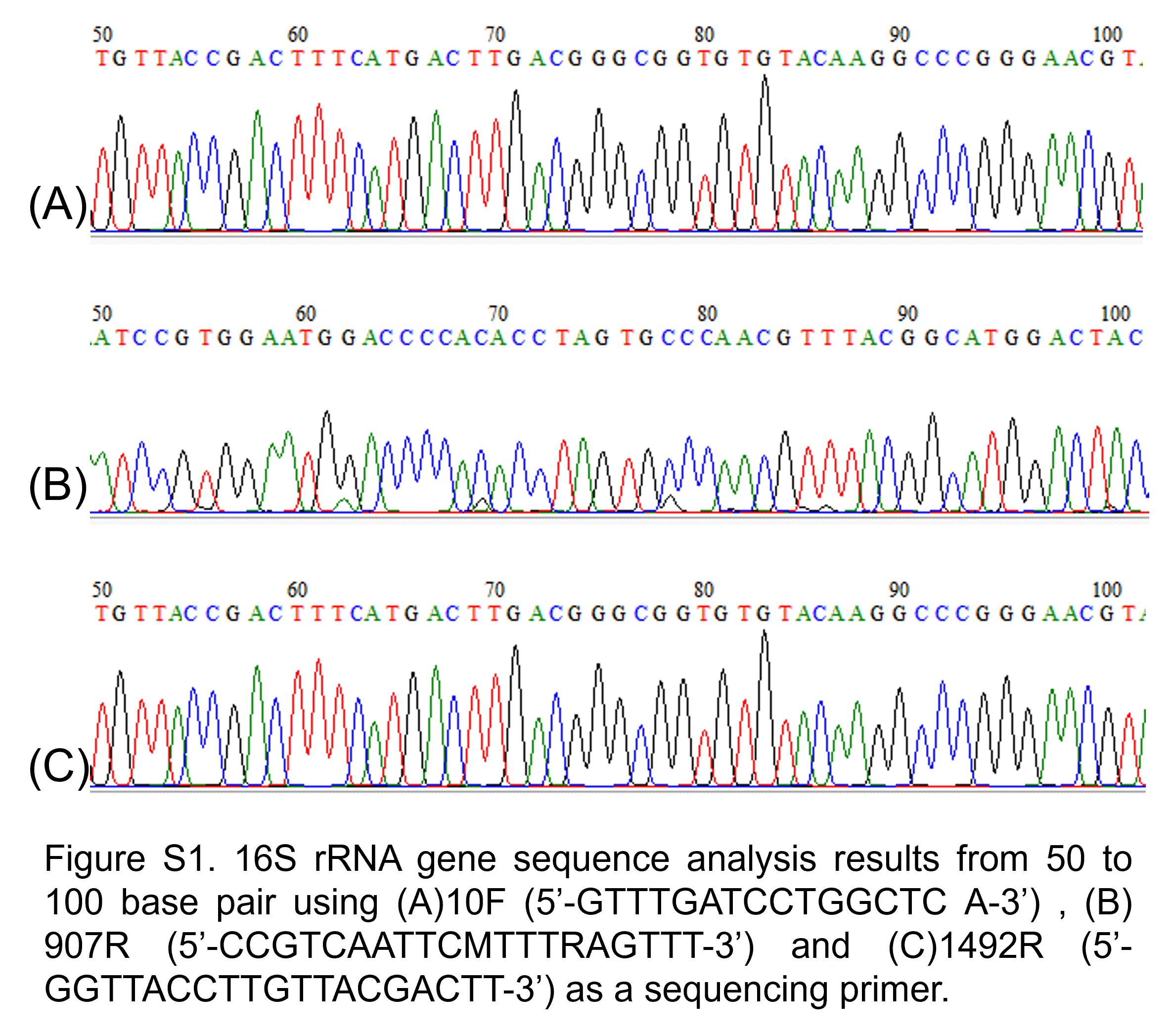

Supplement: Supplementary file 1 [file microorganisms-10-02051-s001.zip › Supplementaly data/FigureS1 16S rRNA gene sequence reults.png]

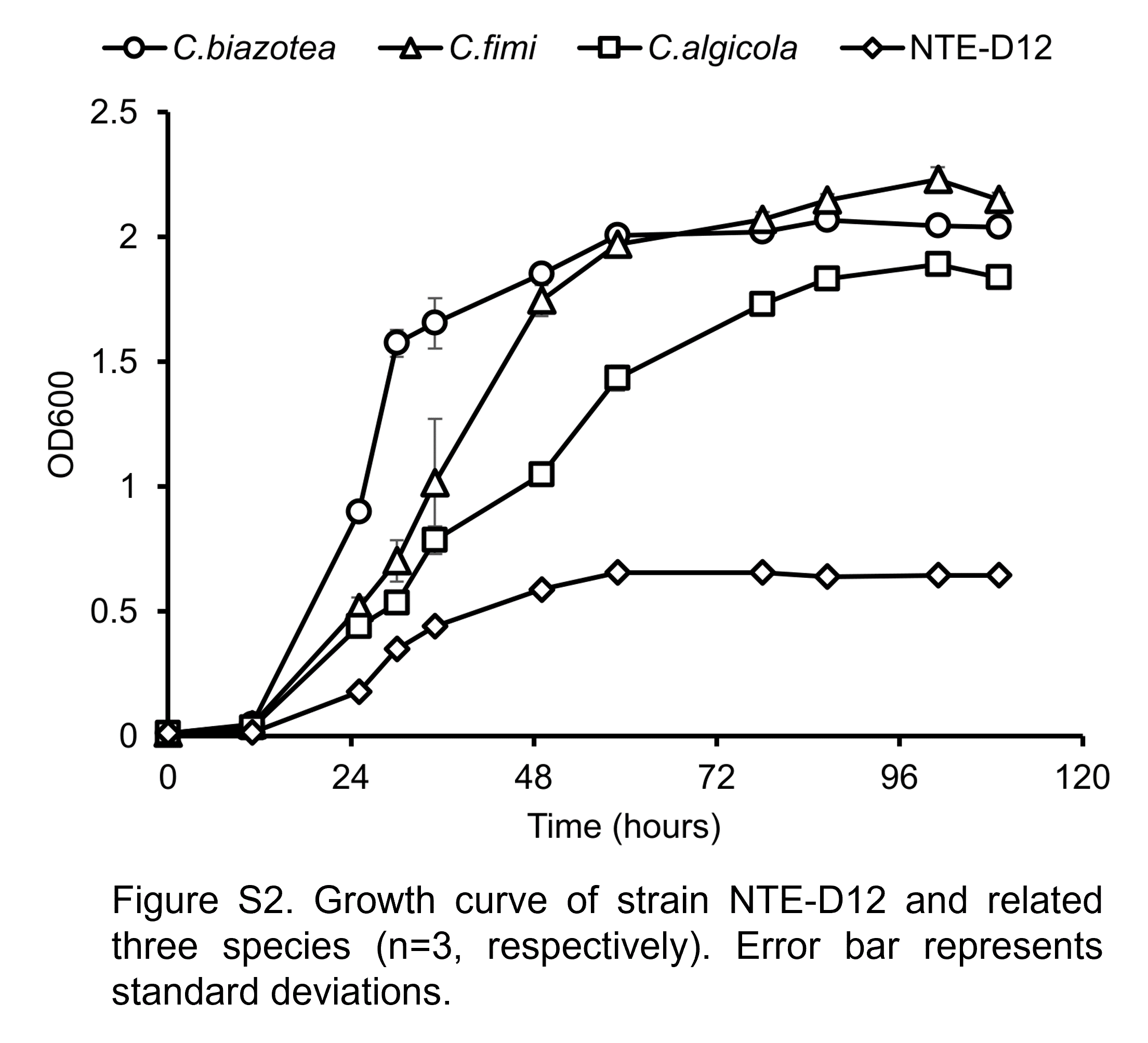

Supplement: Supplementary file 1 [file microorganisms-10-02051-s001.zip › Supplementaly data/FigureS2 Growth curve of strain NTE-D12 and related three species.png]
